# A hybrid deep learning network for automatic diagnosis of cardiac arrhythmia based on 12-lead ECG

**DOI:** 10.1038/s41598-024-75531-w

**Published:** 2024-10-18

**Authors:** Xiangyun Bai, Xinglong Dong, Yabing Li, Ruixia Liu, Henggui Zhang

**Affiliations:** 1 School of Computer Science and Technology, Xi’an University of Posts and Telecommunications, Xi’an, 710121 China; 2 School of Automation, Xi’an University of Posts and Telecommunications, Xi’an, 710072 China; 3 Xi’an Key Laboratory of Advanced Control and Intelligent Process, Xi’an, 710072 China; 4 School of Physics and Astronomy, The University of Manchester, Manchester, M13 9PL UK; 5 Key Laboratory of Medical Electrophysiology of Ministry of Education and Medical Electrophysiological Key Laboratory of Sichuan Province, Institute of Cardiovascular Research, Southwest Medical University, Luzhou, 646000 China; 6 Beijing Academy of Artificial Intelligence, Beijing, 100000 China

**Keywords:** Electrocardiogram, CNN, BiGRU, Multi-head attention, Cardiac arrhythmia, Feature extraction, MIT-BIH arrhythmia database, Computational biology and bioinformatics, Physiology, Biomedical engineering, Computational science, Computer science

## Abstract

Cardiac arrhythmias are the leading cause of death and pose a huge health and economic burden globally. Electrocardiography (ECG) is an effective technique for the diagnosis of cardiovascular diseases because of its noninvasive and cost-effective advantages. However, traditional ECG analysis relies heavily on the clinical experience of physicians, which can be challenging and time-consuming to produce valid diagnostic results. This work proposes a new hybrid deep learning model that combines convolutional neural network (CNN) and bidirectional gated recurrent unit (BiGRU) with multi-head attention (CBGM model). Specifically, the model consists of seven convolutional layers with varying filter sizes (4, 16, 32, and 64) and three pooling layers, respectively, while the BiGRU module includes two layers with 64 units each followed by multi-head attention (8-heads). The combination of CNN and BiGRU effectively captures spatio-temporal features of ECG signals, with multi-head attention comprehensively extracted global correlations among multiple segments of ECG signals. The validation in the MIT-BIH arrhythmia database achieved an accuracy of 99.41%, a precision of 99.15%, a specificity of 99.68%, and an F1-Score of 99.21%, indicating its robust performance across different evaluation metrics. Additionally, the model’s performance was evaluated on the PTB Diagnostic ECG Database, where it achieved an accuracy of 98.82%, demonstrating its generalization capability. Comparative analysis against previous methods revealed that our proposed CBGM model exhibits more higher performance in automatic classification of arrhythmia and can be helpful for assisting clinicians by enabling real-time detection of cardiac arrhythmias during routine ECG screenings.

**Supplementary Information:**

The online version contains supplementary material available at 10.1038/s41598-024-75531-w.

## Introduction

Cardiovascular diseases have emerged as a primary cause of death globally among non-communicable diseases. When individuals are afflicted with cardiovascular diseases^1^, the electrocardiogram (ECG) reveals anomalous signals. Following the standards set by the American Medical Instrumentation Association, ECG data is comprehensively categorized into distinct types, including Normal Sinus Rhythm, Atrial Premature Contraction, Ventricular Premature Contraction, Left Bundle Branch Block, and Right Bundle Branch Block^2^. In instances where ECG signals exhibit other classifications, indicating irregular cardiac rhythms, further thorough examination and treatment are imperative^3^. This classification furnishes vital insights for medical professionals, aiding them in the diagnosis and treatment of cardiovascular diseases. It is worth noting that ECG, as a non-invasive and cost-effective diagnostic tool^4^, assumes a pivotal role in the diagnosis of cardiovascular diseases.

The ECG stands as the most widely employed diagnostic tool in cardiology^5^. Understanding and interpreting ECG recordings are crucial for enhancing diagnostic accuracy and ensuring timely management of patients experiencing arrhythmias and cardiac incidents. However, diagnostic outcomes heavily rely on the clinician’s extensive clinical diagnostic experience and may be influenced by uncertainties associated with certain cardiovascular conditions. In addition, when continuous monitoring of a patient’s dynamic ECG is required for an extended period, clinicians may face the challenge of misdiagnosis or overlooking cases due to prolonged and continuous work hours^6^. Therefore, it is crucial to develop an optimal classifier for real-time detection of cardiac arrhythmias.

Over the past decade, researchers have developed various machine learning algorithms to compile diagnostic results and diagnose various arrhythmic diseases from ECG. Algorithms such as Support Vector Machine (SVM)^7^, k-Nearest Neighbors^8^, Principal Component Analysis^9^, and Adaptive Backpropagation Neural Network^10^ have shown promising results. Traditional methods for ECG signal classification heavily rely on feature engineering and conventional machine learning algorithms. However, these methods have limitations in handling the nonlinearities of heavy loads and automatically extracting features. Traditional approaches may struggle with manual feature extraction due to susceptibility to subjective factors, and they might fail to capture subtle changes in ECG signals, leading to incorrect diagnoses.

To overcome these limitations, deep learning techniques have been increasingly employed for ECG signal classification due to their ability to automatically extract meaningful features from raw data and handle complex nonlinear relationships. Convolutional neural network (CNN)^11^, recurrent neural network (RNN)^12^, long short-term memory (LSTM)^13^, gate recurrent unit (GRU)^14^ and residual neural network^15^ have all been explored for this purpose. CNNs excel at capturing local spatial features but may miss temporal dependencies in time-series data^16^. While RNN-based models such as GRU and LSTM are better suited for time-series data, but GRU is computationally more straightforward, significantly improving training efficiency. GRU can not only learn the temporal dynamics of input data but also selectively remember or forget information in the current memory state, making them popular choices for ECG signal classification. A GRU network for classifying normal and abnormal pulses in ECG can achieve an accuracy of 98.85%^17^ in a binary classification problem. A five-classification algorithm, which added an attention mechanism to the GRU model to enhance the extraction of key information of ECG signals and achieve a classification accuracy of 98%^18^. However, how to design an algorithm that can simultaneously focus on the spatiotemporal features of the ECG signals, so that the model acquires more contextual information and enhances the correlation between the local information, thus improving the arrhythmia classification ability is a problem to be solved.

Therefore, this study aims to investigate a hybrid deep learning model for ECG classification, enabling effectively capture crucial spatiotemporal features of ECG signals to improve the accuracy of arrhythmia classification. To summarize, the primary contributions of this work are as follows:

- In this work, we designed a hybrid CNN-BiGRU model with multi-head attention mechanism (CBGM model) to effectively capture the spatiotemporal features of ECG signals. The CNN module efficiently captures spatial patterns, while the BiGRU component processes temporal dependencies, ensuring a comprehensive understanding of the signal’s dynamics., enabling the model to thoroughly process both spatial and temporal information.
- To further enhance the model’s ability to focus on important spatiotemporal features, we integrated a multi-head attention mechanism. This mechanism allows the model to capture global correlations across different segments of the ECG signals, directing attention to the most relevant features and improving classification performance.
- To illustrate the generalization ability of our CBGM model, we validated the model using two publicly available datasets, the MIT-BIH and PTB ECG datasets.

Experimental results demonstrate that the superior performance of our model in ECG signal classification, surpassing existing methods. This work provides a beneficial exploration for the development and application of the CNN-BiGRU model based on a multi-head attention mechanism in the field of ECG signal classification.

## Methods

**Fig. 1:**
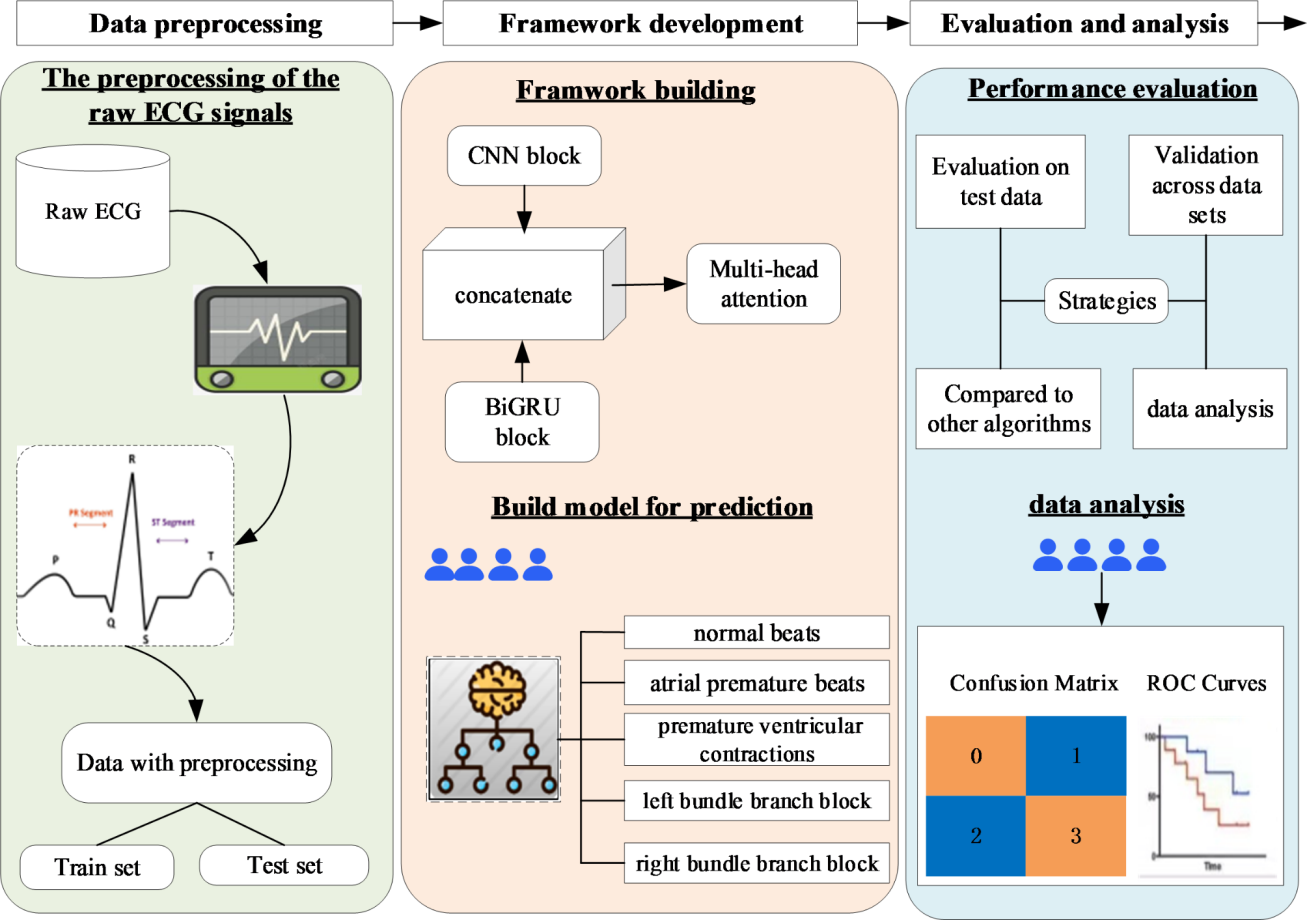
The overview of this study. The whole ECG signals classification process including: preprocessing, feature extraction and classification, and results evaluation and analysis

In summary, the whole process in this work can be divided into 3 steps: preprocessing, feature extraction and classification, and results evaluation and analysis. The overview process is shown in Fig. 1.

### Preprocessing

*Datasets* The MIT-BIH Arrhythmia Database was chosen because it is extensively used in scientific research on cardiac arrhythmias classification. This database encompasses various types of electrocardiogram signals and offers a substantial amount of data. Cardiology experts annotated those rhythm files separately, primarily categorizing them into five types, including normal beats (N), atrial premature beats (A), premature ventricular contractions (V), left bundle branch block (L), and right bundle branch block (R), as shown in Supplement Table S1. This database comprises 48 ECG records, each lasting 30 min. The signal sampling rate is 360 Hz.

In addition, PTB Diagnostic ECG Database was further used to verify the generalization ability of our proposed CBGM model. The database contains 290 records from 549 subjects. Each record includes 15 simultaneously measured signals: the traditional 12 leads and 3 Frank leads. Each signal is digitized at a rate of 1000 samples per second, with a resolution of 16 bits within a range of ± 16.384 mV.

*Denoising* ECG signals are characterized by their weak, low-amplitude, low-frequency, and stochastic nature, making them susceptible to various forms of noise interference. In this work, a combination of Kalman filtering and wavelet transform is used to preprocess the raw ECG signals. Firstly, the original ECG signal is decomposed into multiscale wavelet coefficients through wavelet decomposition. Subsequently, these coefficients undergo dynamic estimation and filtering using Kalman filtering. Finally, the filtered ECG signal is reconstructed through wavelet reconstruction. The detailed experimental steps are outlined in the Online Supplement.

*R peaks detection* Detection of the QRS waveform is a necessary prerequisite for segmenting and classifying the denoised ECG signal^19^. In this work, during the process of decomposing ECG signals using wavelet transform, the signal is decomposed into various sub-signals, concentrating the energy of QRS waveforms in the high-frequency band. Post wavelet transform, we identify regions with concentrated energy to locate the QRS waveforms. Typically, the QRS waveforms exhibit prominent energy concentration within the high-frequency sub-signals post wavelet transformation. Leveraging these concentrated energy positions, combined with thresholds or other criteria, facilitates the detection and labeling of QRS waveforms. We set the threshold at 60% of the signal amplitude. This threshold is designed to identify R waves significantly higher than the average signal amplitude. Since R-wave amplitudes are typically much higher than the average amplitude of an ECG signal, setting the threshold at a low level aids in effectively detecting most R waves. The preprocessing result is shown in Supplement figure S1.

### Feature extraction and classification

The proposed CBGM model in the article extracts spatial features from the electrocardiogram (ECG) signals using convolutional layers, integrates BiGRU layers to maintain dynamic memory of the extracted features, and finally introduces a multi-head attention mechanism to enhance the extraction of crucial ECG signal features. A detailed description of each layer is as below:

*CNN* CNN is a form of deep learning algorithm inspired by the functioning of biological neural systems like the human brain^20^. CNNs can progressively learn the spatial hierarchy of data by memorizing both high-level and low-level patterns. Typically, a CNN comprises three types of layers: convolutional layers, pooling layers, and fully connected layers. The convolutional and pooling layers perform feature extraction and dimensionality reduction, respectively, while the fully connected layers map the extracted features to predict the final output^21^. Regarding the architecture of CNN layers, each layer feeds its output to the next. As information travels through the network, the layer outputs become increasingly complex. This process, known as training, aims to minimize the difference between network predictions and ground truth labels by employing optimization algorithms such as backpropagation and gradient descent. The formula is expressed as follows:

1 $$y(t)=x(t)*w(t)=\int\limits_{{ - \infty }}^{\infty } {x(\tau )w(\tau )} d\tau$$

Here, *x* represents the input, the parameter *w* represents the kernel or filter, and the result y is commonly referred to as the feature map. The convolutional layer is a crucial component of CNNs, responsible for generating feature maps by computing the convolution of its input with these filters.

*BiGRU* BiGRU is a bidirectional recurrent neural network model composed of two parts: the forward GRU and the backward GRU. BiGRU processes the input sequence both forward and backward, then concatenates the results from both directions to obtain a more comprehensive semantic understanding. Compared to GRU, BiGRU is more adept at handling sequential data. Its bidirectional structure enables the network to simultaneously consider past and future information, thereby enhancing its comprehension of context and patterns within time series data, providing robust support for accurate classification of electrocardiogram signals. The detailed formulas’ descriptions of the BiGRU model are shown in the Online Supplement.

*Multi-head attention mechanism* The attention mechanism computes the probability distribution of attention to highlight the influence of specific key inputs on the output, further capturing crucial information within sequences, thereby optimizing the model and enabling more accurate judgments.

The principle of the multi-head mechanism involves mapping query (*Q*), key (*K*), and value (*V*) into different subspaces, where each space performs self-attention computation independently without interference^22^. Eventually, the outputs from each subspace are concatenated, enabling the model to capture more contextual information within the sequence and enhancing its feature representation capacity. Simultaneously, as an ensemble, it helps prevent overfitting. The multi-head attention mechanism, as shown in the equation, depicts each attention mechanism function responsible for a specific subspace in the final output sequence. Each subspace operates independently, and the outputs from multiple subspaces are concatenated, followed by feeding into a fully connected layer to obtain the ultimate feature output. The attention mechanism within each head is defined as follows:

2 $$hea{d_i}=Attention(Q{W^Q},K{W^K},V{W^V})=Soft\hbox{max} \left[ {\frac{{QW_{i}^{Q}{{(KW_{i}^{K})}^T}}}{{\sqrt {{d_k}} }}} \right]VW_{i}^{V}$$

where *W*_*i*_^*Q*^, *W*_*i*_^*K*^, and *W*_*i*_^*V*^ are learning matrices.

3 $$MultiHead(Q,K,V)=concat(hea{d_1},hea{d_2}, \cdot \cdot \cdot ,hea{d_h}){W_o}$$

where *Q*, *K* and *V* are matrices representing the input electrocardiogram signals. *W*_*o*_ is the learning matrix. *h* represents the number of subspaces. Due to the properties of the Softmax function, when the input values are extremely large, the function tends to converge towards a region of very small gradients. Therefore, the scaling factor $1/\sqrt {{d_k}}$is used to counteract this effect.

**Fig. 2:**
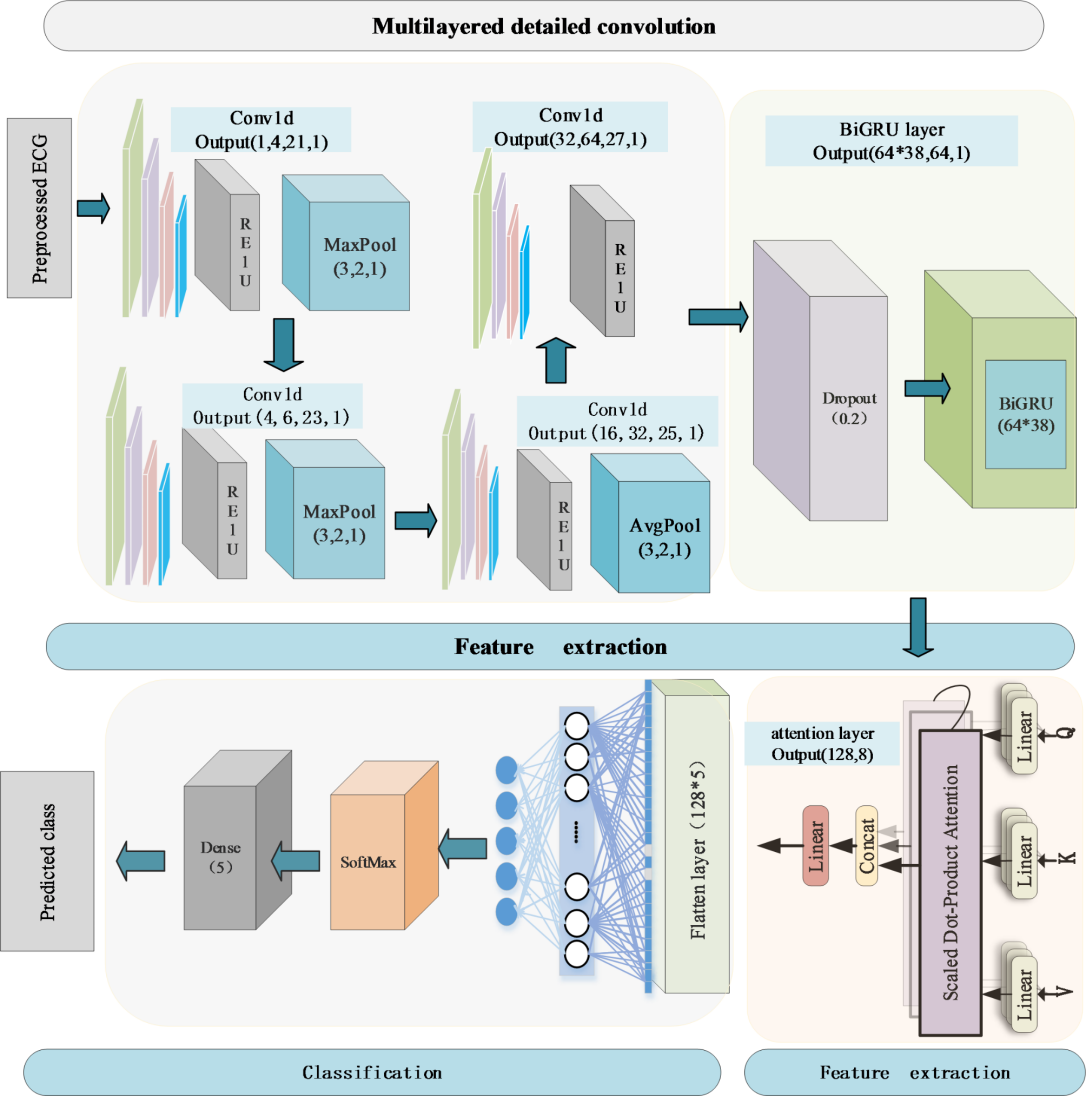
Schematic diagram of the proposed CBGM model. From multilayered detailed convolution with CNNs and BiRGRU layer to multi-head attention based feature extraction and classification

*Proposed CBGM model*. In this work, we propose a CNN-BiGRU model with multi-head attention (CBGM model) that extracts spatial features from ECG signals using convolutional layers, integrates BiGRU layers to maintain dynamic memory of the extracted features, and finally introduces a multi-head attention mechanism to enhance the extraction of crucial ECG signal features, as shown in Fig. 2. The CBGM model that takes ECG pulses as input and categorizes them into five different classes: N, A, V, L, and R. Specifically, we first detected the R peaks in all signals. Then, each pulse was defined as 150ms before and 150ms after the R peak. Regarding the architecture of the model, an input layer was followed by 13 hidden layers and an output layer. The input layer received one-dimensional ECG signals with a length of 300 samples. Initially, the signal passed through a CNN section consisting of three triplets, each comprising a convolutional layer, batch normalization layer, and max-pooling layer. These triplets were placed sequentially in the architecture to facilitate feature extraction. Simultaneously, there was dimensionality reduction as the input traversed deeper into the network. Subsequently, the output was fed into the BiGRU layer, responsible for recognizing and memorizing long-term dependencies between data.

The BiGRU module comprises a BiGRU layer, a fully connected layer, and a dropout layer, with the output layer predicting one of the five classes for each input ECG pulse. The BiGRU network captures long-term dependencies in sequential data, crucial for handling time-series data like ECG signals. We introduced a multi-head attention mechanism on the output of the BiGRU layer. This mechanism dynamically adjusts the weights of the signals based on the importance of each timestep, allowing the model to focus more on the moments crucial for the classification task. The introduced attention mechanism enables the model to concentrate on key features in the ECG signals related to cardiac arrhythmias, making our model more robust compared to traditional models. After feature extraction, we flatten the features and feed them into a fully connected layer, followed by a softmax layer for classification, resulting in the final prediction. The detailed parameter settings for the proposed model are shown in Supplement Table S2.

We focused on optimizing the learning rate, batch size, and the number of training epochs during hyperparameter tuning. Starting with a learning rate of 0.001 and adjusted it based on observed performance. A batch size of 128 was chosen to balance processing time and model stability. The number of training epochs was set to 100, with early stopping based on validation performance to prevent overfitting. Initial training with default hyperparameters provided a baseline, after which we used grid search to test various learning rates (0.01, 0.001, 0.0001), batch sizes (32, 64, 128), and model architectures. Cross-validation ensured robust evaluation. The optimal configuration was a learning rate of 0.001 and a batch size of 128. In our proposed CBGM model, with the combination of convolutional layers, pooling layers, a GRU layer, and a multi-head attention mechanism, significantly improved the capture of time-series data, enhancing performance in processing ECG signals. All experiments were conducted in a Python 3.9 environment using the PyTorch package, on a desktop equipped with an Intel Core i5-9600 K 3.70 GHz CPU, 16 GB RAM, and an 8 GB NVIDIA GeForce RTX 2070 GPU.

### Performance evaluation method

The performance of our proposed model was evaluated using various metrics including precision, specificity, F1 score, sensitivity, and accuracy, which are as follows:

4 $$precision=\frac{{TP}}{{TP+FP}}$$

5 $$Accuracy=\frac{{TP+TN}}{{TP+TN+FP+FN}}$$

6 $$specificity=\frac{{TN}}{{TN+FP}}$$

7 $$F1 - score=2 \times \frac{{recall \times precision}}{{recall+precision}}$$

where *FP*, *TP*, *FN*, and *TN* respectively represent “False Positive,” “True Positive,” “False Negative,” and “True Negative.

## Result analysis

### Classification performance on MIT-BIH arrhythmia database

Firstly, the test set was employed to assess the effectiveness of our proposed model. The results of ten runs using the proposed CBGM model are listed in Table 1. It is noteworthy that the 10th experiment outperformed the average in all evaluation metrics. Furthermore, the 7th experiment achieved the highest accuracy and F1 score. Our proposed CBGM model has achieved a mean accuracy of 99.41%.

**Table 1:** Classification scores on MIT-BIH database

No.	Accuracy (%)	Precision (%)	Specificity (%)	F1-Score (%)
1	99.43	99.52	99.90	99.31
2	99.46	98.95	98.73	97.42
3	99.37	98.93	99.93	98.96
4	99.35	99.15	99.96	99.13
5	99.26	98.94	99.74	99.32
6	99.38	99.23	99.65	98.99
7	99.55	99.42	99.47	99.65
8	99.26	99.13	99.52	97.69
9	99.35	98.97	99.32	99.35
10	99.43	99.17	99.84	99.26
Mean	99.41	99.15	99.68	99.21

**Fig. 3:**
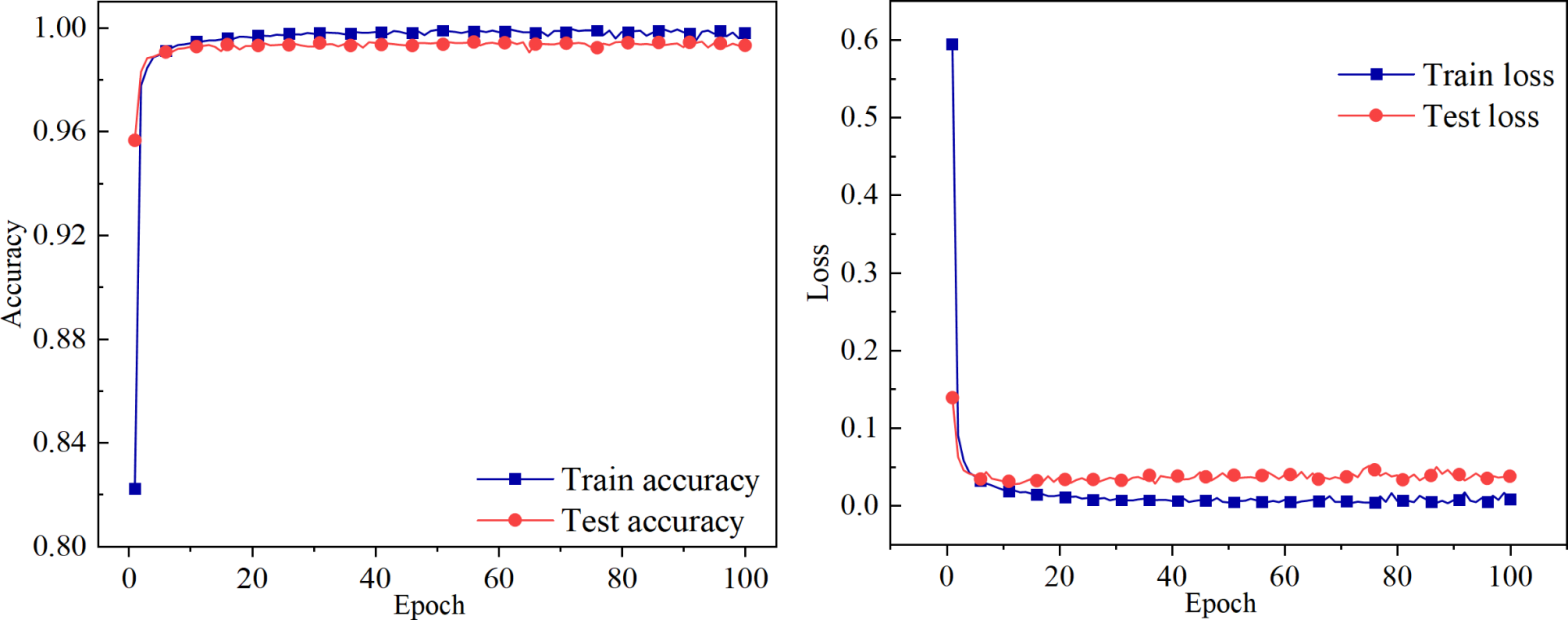
Training/Testing accuracy and loss as a function of epochs

In addition, a comparison between the training/testing accuracy and loss are shown in Fig. 3. The testing accuracy gradually grows in tandem with the training accuracy with the proposed model, indicating a stable model performance without overfitting or underfitting. The final training accuracy is 99.82% and the testing accuracy is 99.42%. This alignment between training and testing accuracies suggests that the model effectively learns from the training data and generalizes well to unseen testing data. The consistency between the two curves throughout the training process underscores the robustness of the proposed model in capturing the underlying patterns in the data without excessively memorizing the training samples or failing to capture the underlying structure of the data.

**Fig. 4:**
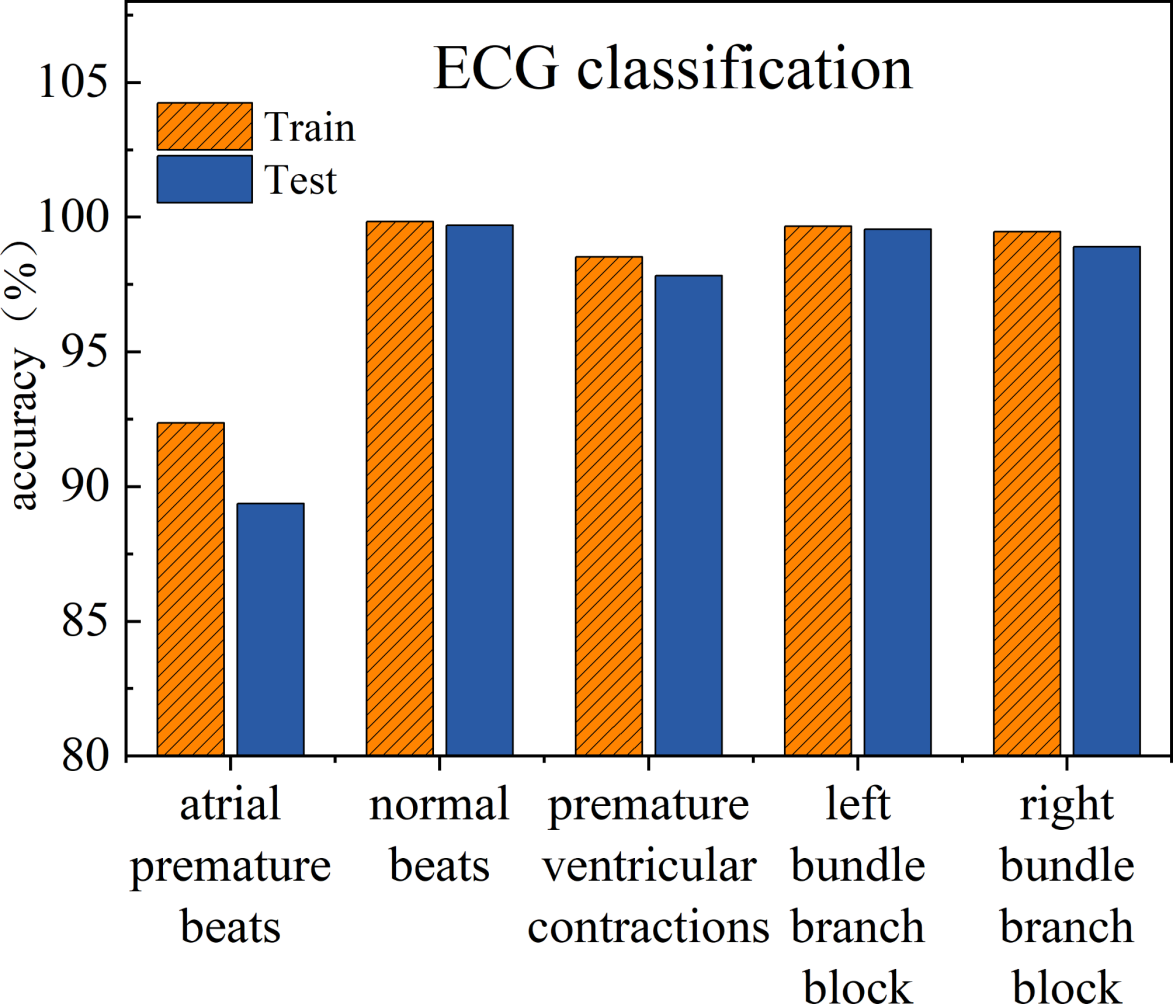
Classification performance of the CBGM model on five types of ECG signals in training and testing conditions

The training and testing performance of the CBGM model on each type of ECG signals are also shown in Fig. 4. The difference in accuracy between the training and testing sets for the five types of electrocardiogram signals does not exceed 3%. The Left bundle branch block achieved the highest training and testing accuracy (99.65% and 99.54% respectively), indicating the best classification of this disease.

**Fig. 5:**
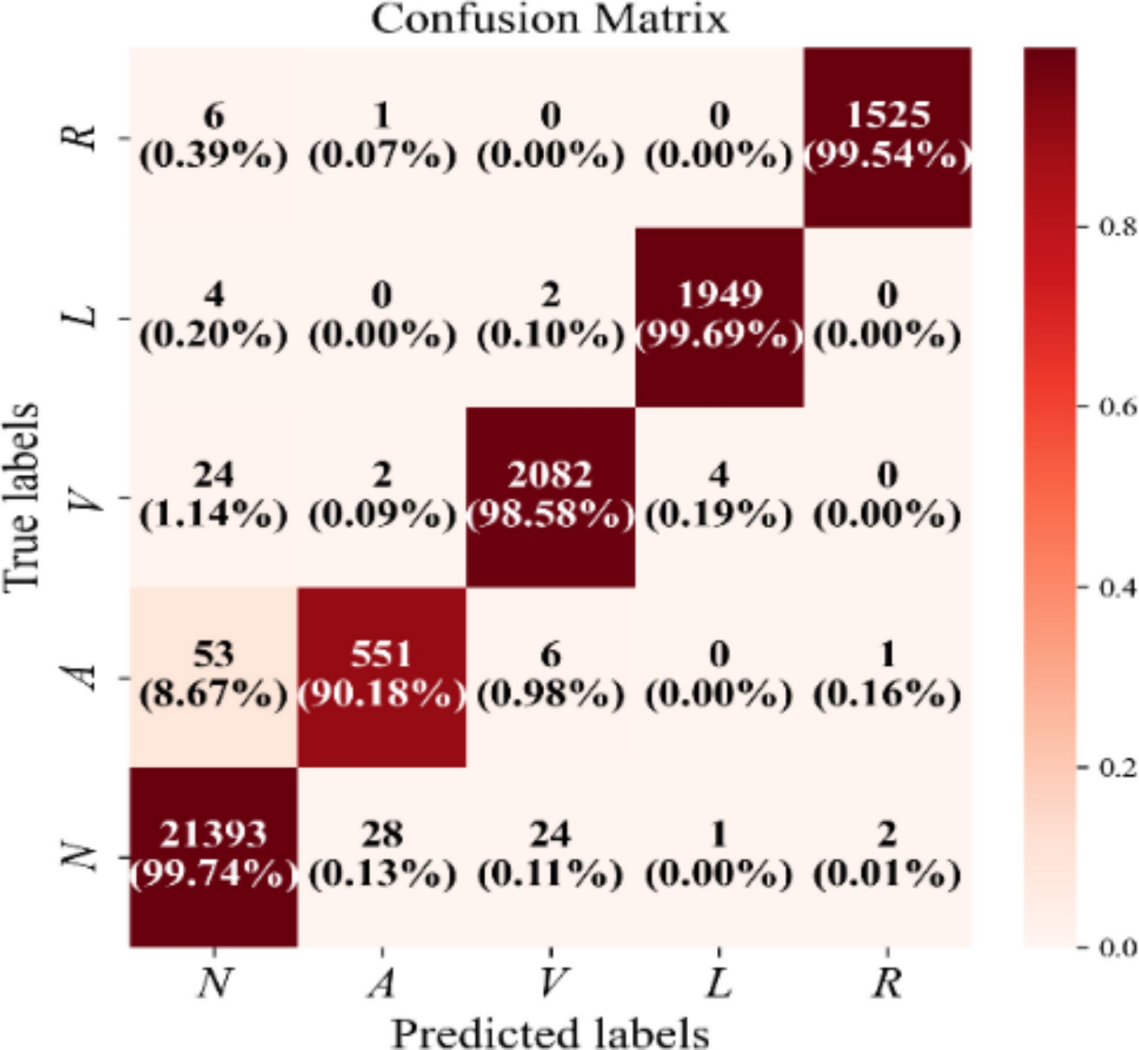
Confusion matrix of the CBGM model for five types of ECG signals

To comprehensively assess the classifier, we further calculated the confusion matrix between the correctly predicted class and the actual class, as shown in Fig. 5. The proposed CBGM model has achieved 99.74% accuracy on N class, 99.69% on L class, 99.58% on V class, 99.54% on R class and 90.18% on A class. The lowest accuracy in categorizing atrial premature beats (A) may be related to its smaller data size compared to the other categories. However, our model has been predicted a total number of 27,500 samples out of 27,658 samples which indicates that only 156 samples (approximately 0.564%) have been misclassified, suggesting that our model in general has better overall classification ability.

The performance comparison between our CBGM model and some state-of-the-art ECG signal classification model are shown in Table 2. Our model has the highest average accuracy, precision and F1-score, which attributed to its effective extraction of spatio-temporal features and improved classification performance. The specificity of our model was 98.68%, which is not the highest but better than most models. Overall, our model has the best classification ability compared to other models.

**Table 2:** Model detection performance comparison on MIT-BIH dataset

References	Model description	Average accuracy(%)	Precision (%)	Specificity (%)	F1-Score (%)
Kachuee et al. ^23^	1D-CNN	93.42	94.30	92.15	93.43
Tippannavar et al. ^24^	2D-CNN	94.30	98	96.25	97.3
Xu et al.^25^	CNN + BiLSTM	95.90	96.34	94.97	94.46
Ozaltin et al. ^7^	CNN + SVM	94.67	96	100	95.05
Sun et al.^26^	CNN + RNN	90.64	90	82.14	92.76
Wang et al.^27^	CNN + BGcsSE	98.8	97.2	99.1	97.8
Our CBGM model	CNN + BiGRU + attention	99.41	99.15	98.68	99.21

**Fig. 6:**
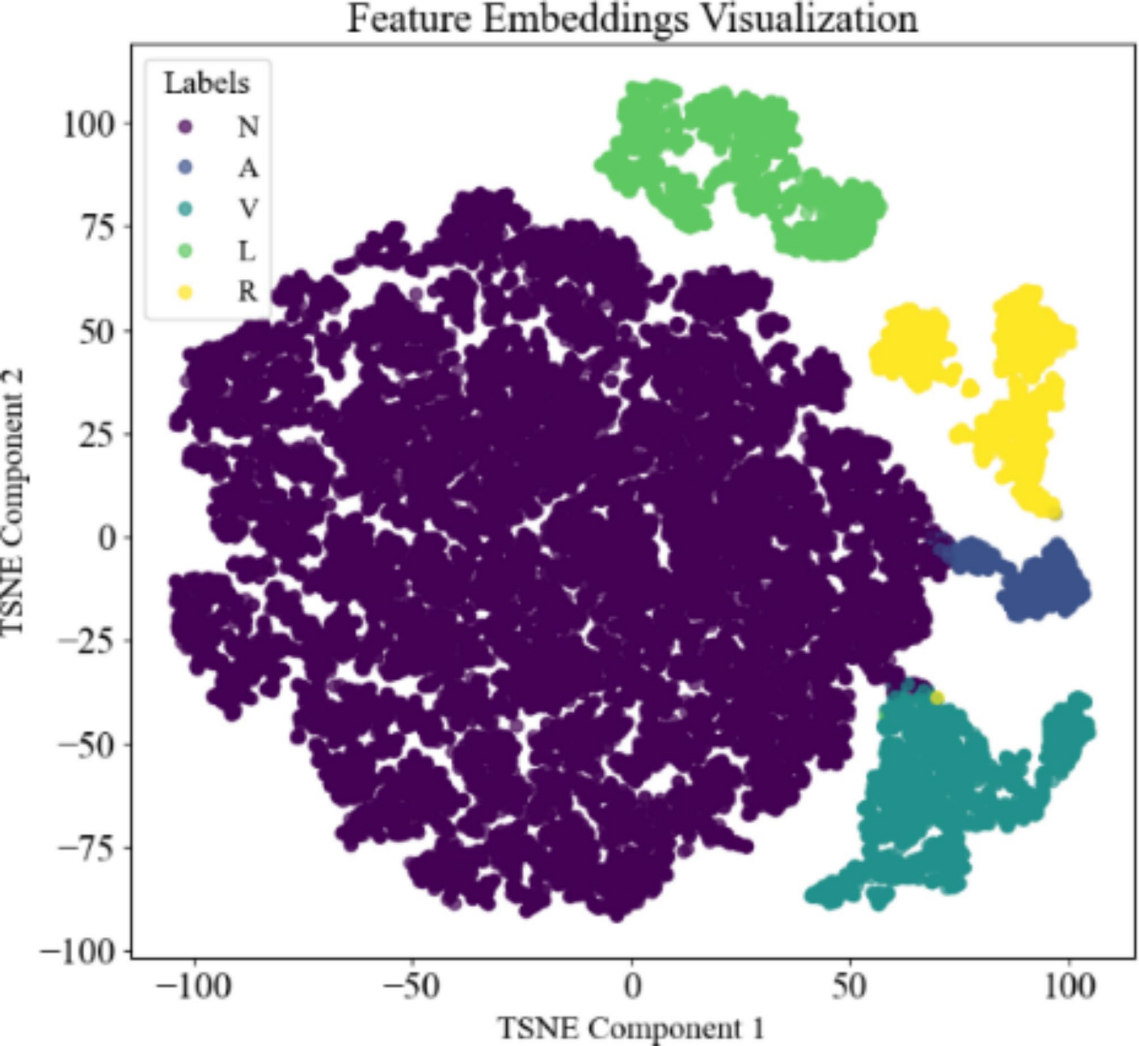
The feature embeddings visualization of five types of ECG signals

In addition, dimensionality reduction techniques was used to project the high-dimensional feature embeddings into the low-dimensional space and visualize them to better show the classification performance of the model. This helps to show the distribution of different categories of data in the feature space and thus validate the effectiveness of the model in feature extraction. As shown in Fig. 6, the feature embeddings visualization displays the distribution of feature embeddings generated by the model for five types of ECG signals (labeled N, A, V, L and R). Different colored points represent different labels, and the distance between points reflects the similarity of features. The figure clearly shows that distinct clusters are formed for each label, indicating that the model effectively distinguishes between different categories. Specifically, the embeddings for label N form a large and tight cluster, whereas the embeddings for label R, though more dispersed, still form a recognizable group. There is a noticeable separation between clusters of different labels, especially between labels N and R. This separation suggests that the model has learned to create unique feature spaces for each category. Additionally, there are some points that do not fit neatly into any cluster, which may represent potential outliers or misclassifications. These outliers provide opportunities for further investigation and improvement of the model’s performance.

### Model generalization capability

The generalization ability is another important aspect to evaluate the performance of a model. Therefore, we employed our CBGM model on the PTB dataset to validate its robustness as previous studies^28^. The preprocessing method for the PTB dataset is the same as that described in the Methods section. And the dataset was split into a training set and a test set at a ratio of 7:3. As shown in Table S3 in the Online Supplement, although the overall classification of our model on the PTB dataset is not as good as the MTI dataset, it outperforms the classification results of other state-of-the-art models on the PTB dataset. Our model with the highest average accuracy, precision and F1-score of 98.82%, 98.33% and 97.31% respectively. The specificity of our model is 96.52%, which is not the highest, but better than most models in Table S3. Overall, our model has the best classification results compared to other models.

**Fig. 7:**
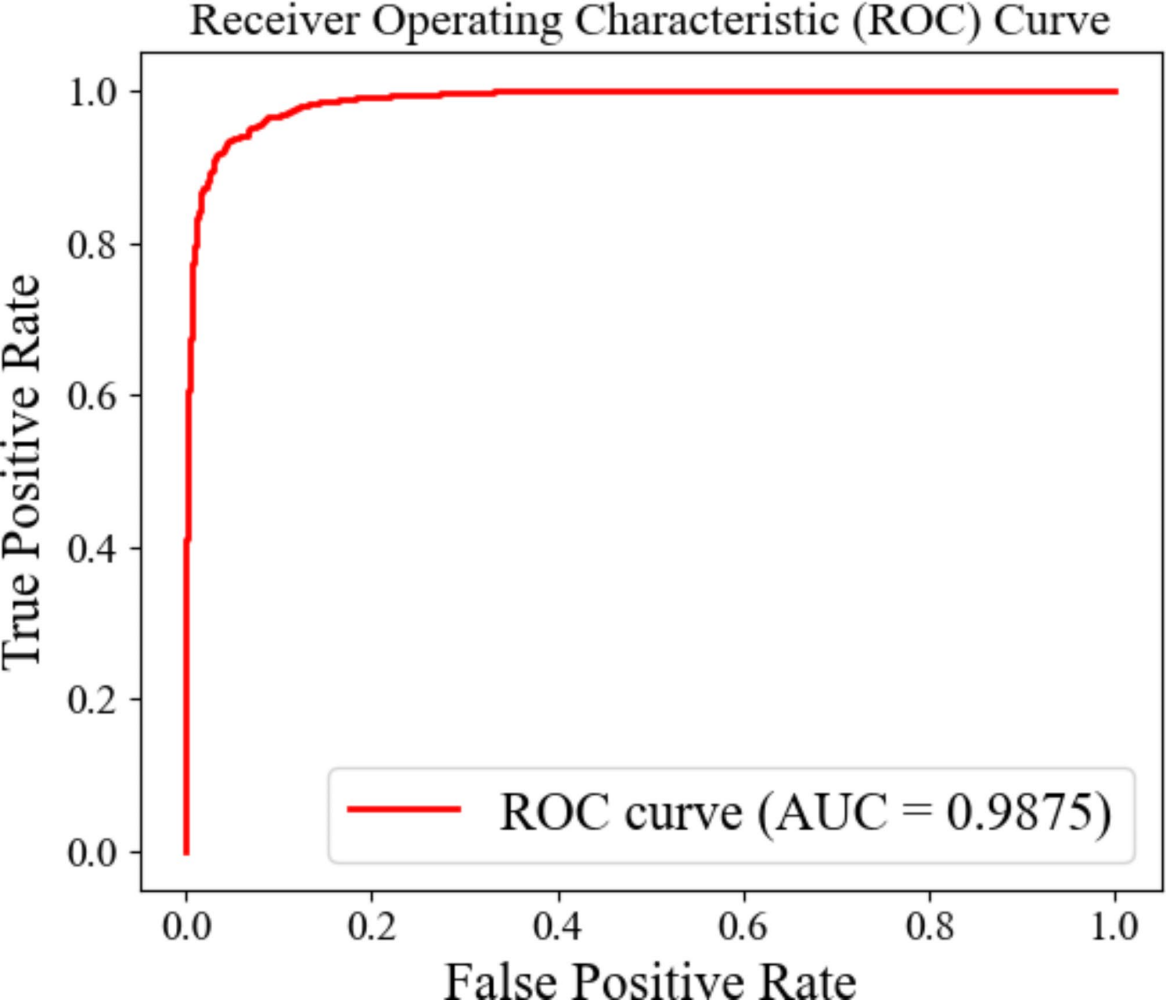
Classification renderings on the PTB dataset

To better understand the performance of our model, we plotted the ROC curve. In Fig. 7, the curve is convex toward the top-left corner, indicating excellent performance of the model across various thresholds. With an AUC value of 0.9875, suggesting that the model can accurately distinguish between positive and negative samples, demonstrating exceptional predictive and generalization capabilities.

## Discussion

Detecting abnormal ECG signals through artificial intelligence algorithms for automatic classification and identification of cardiac diseases has become a crucial task. This study implements a classification approach for ECG signals based on a CNN-BiGRU model with multi-head attention mechanism. During training process on MIT-BIH dataset, the proposed CBGM model achieved an accuracy of 99.41%, F1 score of 99.21%, precision of 99.15%, and specificity of 99.68%. When we transferred the model trained on the MIT-BIH dataset to validate it on the PTBDB dataset, we found the model’s performance to be quite satisfactory, with an accuracy of 98.82%. We compared our model architecture with various state-of-the-art classifiers to demonstrate the superior performance of our proposed model in classification. Particularly, the comparisons in Table 2 and in Supplement Table S3 fully illustrate the practicality of our approach.

In our proposed CBGM model, the multiple attention mechanism is introduced which involves distributing learning across different subspaces from the original attention mechanism to acquire features from various positions at multiple levels. However, the classification performance does not necessarily improve with an increase in the number of subspaces. Having too many subspaces might lead to overfitting, adversely impacting the model’s performance. To address this issue, this work conducted three comparative experiments, denoted as heads-2, heads-4, heads-8 and heads-16, altering only the number of subspaces in the feature enhancement part of the proposed model. The results in Supplement figure S2 show that CBGM model with 8 heads worked best and based on this model the experimental results are shown in the ‘Result analysis’ section.

In addition, we conducted an ablation study on the model proposed in this paper to thoroughly evaluate its performance, as shown in Table 3. Removing either the BiGRU layers or the attention layers resulted in a decrease in classification performance, leading to a decrease in accuracy by about 7% and 9%, respectively. Overall, the utilization of BiGRU layers and attention layers enhanced the classification performance in our CBGM model.

**Table 3:** Ablation results of the CBGM model

BiGRU layer	Attention layer	Accuracy	Precision	Specificity	F1-Score
√		92.35 ± 0.82	91.15 ± 0.74	95.42 ± 1.24	90.24 ± 1.43
	√	90.23 ± 1.13	88.14 ± 0.84	92.36 ± 1.17	89.68 ± 1.58
√	√	99.41 ± 0.15	99.15 ± 0.37	99.68 ± 0.25	99.21 ± 1.79

By employing the multi-head attention mechanism, the CBGM model dynamically focuses on different parts of the input data to capture critical information. Specifically, by passing the output of the BiGRU layer to the multihead attention layer (as shown in Fig. 2), the input *x* is passed through three different layers of linear transformations to generate the *Q*, *K*, and *V* (as shown in formula (3)), respectively. The purpose of these transformations is to map the inputs to different feature spaces so that the attention mechanism can perform computations based on the relationships in each feature space without interference. Eventually, the outputs of all the heads are stitched together and integrated through another linear layer, enabling the model to capture more contextual information within the sequence and enhancing its feature representation capacity. In the ablation study, remove of the attention layer resulted in a significant decrease in accuracy (nearly 7% in Table 3) on the validation set, verifying the effectiveness of the multi-head attention mechanism in improving the performance of the model.

**Fig. 8:**
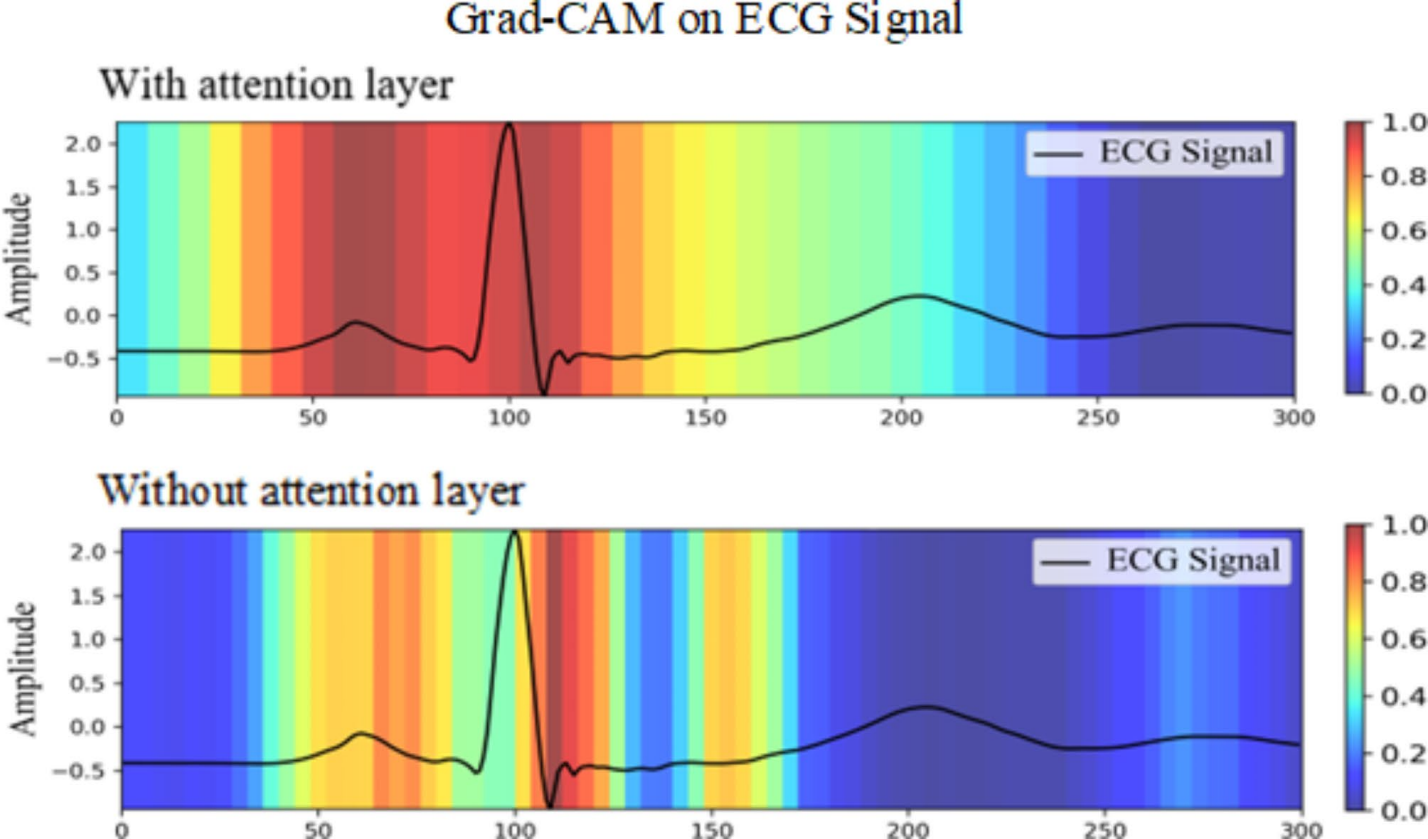
The Grad-CAM comparation of model with and without attention layer.

To further assess the model’s classification performance, we utilize the gradient-weighted class activation mapping (Grad-CAM)^29^ to compare the difference between model with and without attention layer. As shown in Fig. 8, the attention layer significantly increases the model’s focus on relevant features. In the heatmap, red represents the highest focus level, and blue represents the lowest concentration level of the network. It is evident that the model’s attention to QRS complex increases significantly when the attention layer is included. Since this module can alter the weights of key features, and help the neural network classify correctly.

The BiGRU layers are utilized to model sequential data, effectively capturing long-range dependencies within sequences. Simultaneously, CNN is employed to extract spatial features, achieving multi-scale, multi-level feature extraction of input data through hierarchical convolutional operations. This combination enables the model to fully utilize both the temporal and spatial information of the input data, thereby improving its understanding and capturing capabilities of complex data patterns, subsequently enhancing the model’s performance and generalization ability.

The long-term goal of realizing high-performance ECG signal classification models is to enable clinical applications. However, diversity and variable quality of clinical data may affect the generalization ability and accuracy of the model. ECG data collected from different hospitals and devices may vary. An effective solution maybe collect data from multiple centers and devices and perform data cleaning and standardization to improve the consistency and representativeness of the data. Meanwhile, continuously monitor and update the model to ensure its robustness in different data environments are also important. In a clinical setting, ECG analysis needs to be performed in real-time for rapid diagnosis and decision making. Computational resource limitations may affect the deployment and operation of the model. It is necessary to optimize the computational efficiency of the model, simplify the model structure, or adopt edge computing devices to enhance real-time analysis. And conduct detailed performance tests before deployment to ensure the model’s operational efficiency in real applications.

## Conclusion

To solve the current problem of inaccurate and time-consuming in manual diagnosis of arrhythmia from ECG, we developed a new deep learning model for automatic classification of arrhythmia. We successfully constructed a model that integrates convolutional neural networks and bidirectional gated recurrent units while introducing a multi-head attention mechanism to enhance the model’s focus on crucial ECG signal features. Through this hybrid model, we effectively extracted spatial and temporal features, resulting in significant performance improvements in ECG signal classification. This implies the potential deployment of CBGM model in healthcare systems to assist physicians in identifying patients with cardiac arrhythmias, thereby preventing adverse events.

## Electronic supplementary material

Below is the link to the electronic supplementary material.

Supplementary Material 1

## Data Availability

The MIT-BIH arrhythmia database can be found online at MIT-BIH Arrhythmia Database v1.0.0 (physionet.org, https://www.physionet.org/content/mitdb/1.0.0/) and the PTB Diagnostic ECG Database can be found online at PTB Diagnostic ECG Database v1.0.0 (physionet.org, https://www.physionet.org/content/ptbdb/1.0.0/). Data that support the findings of this study can be found at https://pan.baidu.com/s/1SOip1-93soy5I3 × 8vpMDzg with the primary accession code nj81.
