# Supplementary material for: A hybrid deep learning network for automatic diagnosis of cardiac arrhythmia based on 12-lead ECG
Source: Sci Rep. 2024 Oct 18;14:24441. doi: 10.1038/s41598-024-75531-w (PMC11489693; doi:10.1038/s41598-024-75531-w)
Supplement: Supplementary file 1 — Supplementary Material 1 [file 41598_2024_75531_MOESM1_ESM.docx]

**Online Supplement**

**A Hybrid Deep Learning Network for Automatic Diagnosis of Cardiac Arrhythmia Based on 12-lead ECG**

Xiangyun Bai^1^*, Xinglong Dong^1^, Yabing Li^1^, Ruixia Liu^2,3^, Henggui Zhang^4,5,6*^

1. *School of Computer Science and Technology, Xi’an University of Posts & Telecommunications, Xi’an 710121, China*
2. *School of Automation, Xi’an University of Posts & Telecommunications, Xi’an 710072, China*
3. *Xi’an Key Laboratory of Advanced Control and Intelligent Process, Xi’an 710072, China*
4. *School of Physics and Astronomy, The University of Manchester, M13 9PL Manchester, United Kingdom*
5. *Key Laboratory of Medical Electrophysiology of Ministry of Education and Medical Electrophysiological Key Laboratory of Sichuan Province, Institute of Cardiovascular Research, Southwest Medical University, Luzhou, 646000, China*
6. *Beijing Academy of Artificial Intelligence, Beijing, 100000, China*

* Correspondence: henggui.zhang@manchester.ac.uk, baixiangyun@xupt.edu.cn

**Datasets**

The description of classification of ECG signals in MIT-BIH Database is shown in Table S1.

Table S1. Classification of ECG signals in MIT-BIH Database.

| Label | Class | Number of beats |
| --- | --- | --- |
| 0 | N: normal beats | 71273 |
| 1 | A: atrial premature beats | 1950 |
| 2 | V: premature ventricular contractions | 6794 |
| 3 | L: left bundle branch block | 6578 |
| 4 | R: right bundle branch block | 4967 |
| Total |  | 92192 |

**Denoising steps**

Firstly, due to the mixture of noise and signal, ECG signal containing noise is subjected to scale decomposition using wavelet transform to obtain wavelet coefficients at different scales, expressed as:

$W_{j,k}=\int_{-\infty}^{\infty} x(t)\cdot\psi_{j,k}(t)dt$ (1)

Here, *W_j,k_* represents the wavelet coefficients on each scale j and translation k, *ψ_j,k_*(t) represents the wavelet function at scale *j* and translation *k*.

Secondly, we apply Kalman filtering to the wavelet coefficients. At each scale, treat the wavelet coefficients *W_j,k_* as system states and use Kalman filtering for dynamic estimation and filtering. In the prediction step, utilize the dynamic model of wavelet coefficients to forecast the coefficients for the next time step, expressed as:

$\tilde{W}_{j,k}^{+}=F\cdot\tilde{W}_{j,k}$ (2)

Then, in the update step, correct the predicted coefficients using the measured values (original coefficients) and adjust the uncertainty of the coefficients based on the difference between observed and predicted values.

$\tilde{W}_{j,k}^{+}=\tilde{W}_{j,k}^{+}+K\cdot(W_{j,k}-H\cdot\tilde{W}_{j,k}^{+})$ (3)

Where *F* is the state transition matrix, *K* is the Kalman gain, and *H* is the measurement matrix.

Finally, we utilize the wavelet coefficients processed by Kalman filtering for wavelet reconstruction. Reconstruct the filtered ECG signal by combining the filtered wavelet coefficients (*x_filtered_*), represented as:

$x_{filtered}(t)=\sum_{j,k} \tilde{W}_{j,k}^{+}\cdot\psi_{j,k}(t)$ (4)

**Preprocessing result**

The preprocessing of the raw ECG signals is shown in figure S1.

Figure S1. Preprocessing of the raw ECG signals.

**BiGRU**

The formulas for the BiGRU module are as follows:

 （5）

 （6）

$h_{t}=w_{t}\vec{h}_{t}+v_{t}\overleftarrow{h}_{t}+b_{t}$ （7）

Here, GRU(·) represents the non-linear transformation of the input time vector, *w_t_*, *v_t_* denote the weights corresponding to the forward hidden state and backward hidden state of BiGRU at time respectively; *b_t_* represents the bias of the hidden layer state at time *t*.

The parameter settings for our proposed CBGM model are shown in Table S2.

Table S2. Parameter settings for the CBGM model.

| Layer | Type | Number fileter/unit | Kernel Size | Activation function |
| --- | --- | --- | --- | --- |
| 1 | Convolution1D | 300 | 21 | RelU |
| 2 | Convolution1D | 300 | 21 | RelU |
| 3 | Max-pooling | 150 | 3 |  |
| 4 | Convolution1D | 150 | 23 | RelU |
| 5 | Convolution1D | 150 | 23 | RelU |
| 6 | Max-pooling | 75 | 3 |  |
| 7 | Convolution1D | 75 | 25 | RelU |
| 8 | Convolution1D | 75 | 25 | RelU |
| 9 | Avg-pooling | 38 | 3 |  |
| 10 | Convolution1D | 38 | 27 | RelU |
| 11 | Dropout | - | - | - |
| 12 | BiGRU |  | - | Tanh |
| 13 | Attention | 64 | - | - |
| 14 | Flatten | - | - | - |
| 15 | Dense | 5 | - | Relu |

The detection performance comparison with other models on PTB dataset is shown in Table S3.

Table S3. Model detection performance comparison on PTB dataset.

| References | Model description | Average Accuracy(%) | Precision(%) | Specificity(%) | F1-Score(%) |
| --- | --- | --- | --- | --- | --- |
| Labati *et al*.^1^ | 1D-CNN | 91.42 | 90.31 | 91.25 | 92.12 |
| Strodthoff *et al*.^2^ | Resnet | 91.22 | 98 | 96.25 | 97.3 |
| Śmigiel *et al*.^3^ | Few-Shot Learning (FSL) | 95.90 | 96.34 | 94.97 | 94.46 |
| Sepahvand *et al*.^4^ | CNN+LSTM | 94.67 | 96 | 100 | 95.05 |
| Thalluri  *et al*.^5^ | CNN+LSTM+attention | 95.90 | 93.9 | 98.9 | 95.3 |
| Alkhawal *et al*.^6^ | DeepResidual +BiLSTM | 98.7 | 98 | 97.90 | 98 |
| Our CBGM model | CNN+BiGRU +attention | 98.82 | 98.33 | 96.52 | 97.31 |


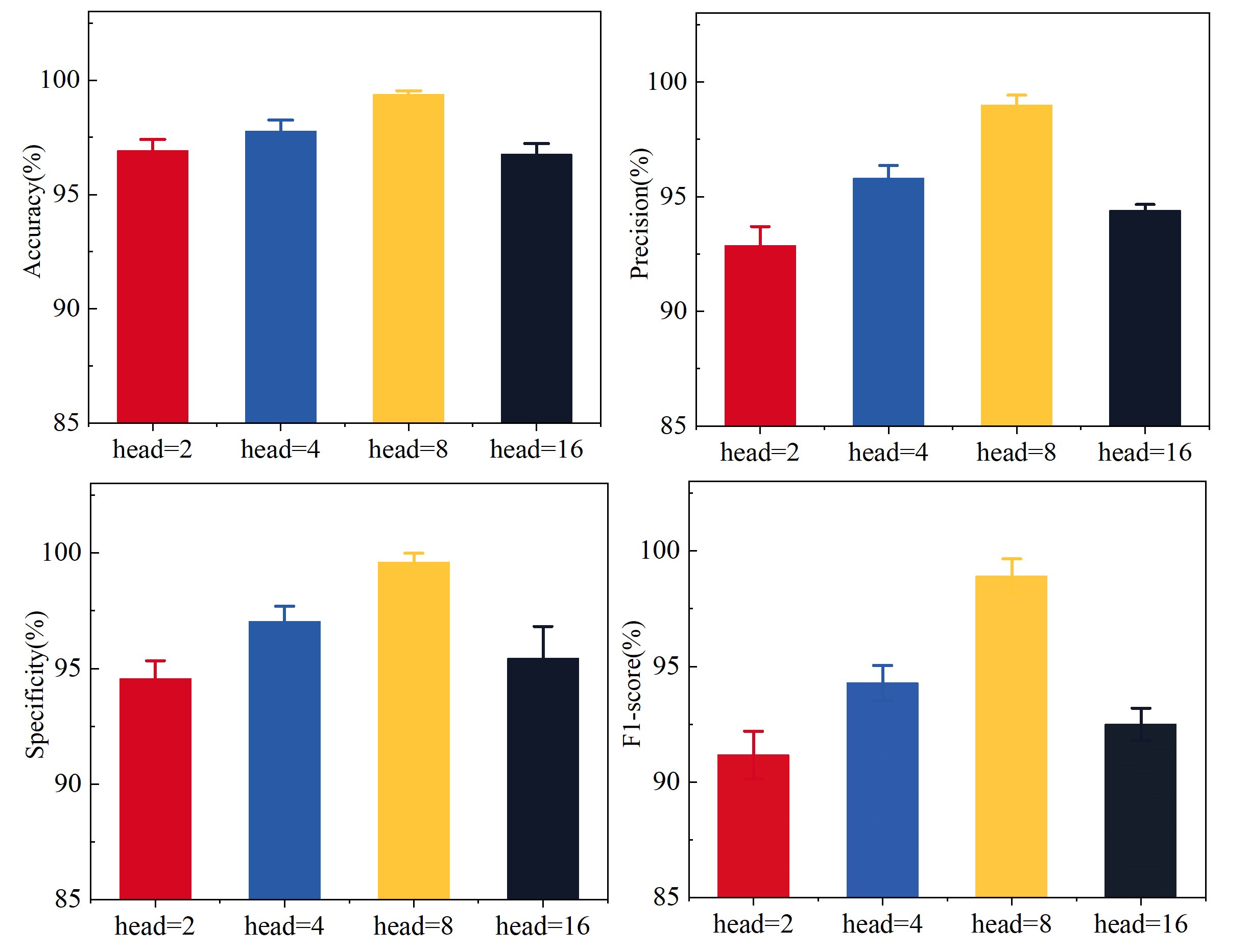


Figure S2. Performance of the CBGM model with different number of heads.

**References**

1 Labati, R. D., Muñoz, E., Piuri, V., Sassi, R. & Scotti, F. Deep-ECG: Convolutional neural networks for ECG biometric recognition. *Pattern Recognit. Lett.* **126**, 78-85 (2019).

2 Strodthoff, N., Wagner, P., Schaeffter, T. & Samek, W. Deep learning for ECG analysis: Benchmarks and insights from PTB-XL. *IEEE J. Biomed. Health. Inf.* **25**, 1519-1528 (2020).

3 Śmigiel, S., Pałczyński, K. & Ledziński, D. Deep learning techniques in the classification of ECG signals using R-peak detection based on the PTB-XL dataset. *Sensors* **21**, 8174 (2021).

4 Sepahvand, M. & Abdali-Mohammadi, F. A novel multi-lead ECG personal recognition based on signals functional and structural dependencies using time-frequency representation and evolutionary morphological CNN. *Biomed. Signal Process. Control* **68**, 102766 (2021).

5 Thalluri, L. N. *et al.* in *2022 7th International Conference on Communication and Electronics Systems (ICCES).* 273-280 (IEEE).

6 Alkhawaldeh, R. S. *et al.* Convolution neural network bidirectional long short-term memory for heartbeat arrhythmia classification. *Int. J. Comput. Intell. Syst.* **16**, 197 (2023).
